# Culturally Tailored Social Media Content to Reach Latinx Immigrant Sexual Minority Men for HIV Prevention: Web-Based Feasibility Study

**DOI:** 10.2196/36446

**Published:** 2022-03-16

**Authors:** Jane J Lee, Joel Aguirre Herrera, José Cardona, Loren Yesenia Cruz, Lésster Munguía, Christopher A Leyva Vera, Gabriel Robles

**Affiliations:** 1 School of Social Work University of Washington Seattle, WA United States; 2 Entre Hermanos Seattle, WA United States; 3 School of Social Work Rutgers University New Brunswick, NJ United States

**Keywords:** social media, eHealth, feasibility, Latinx, immigrant, sexual minorities, gay, homosexual, bisexual, pre-exposure prophylaxis, HIV prevention, HIV, prevention, web-based, internet-based, sexual minority, sexual health, digital health, health technology, web-based health, web-based prevention, health information

## Abstract

**Background:**

Latinx gay, bisexual, and other sexual minority men are disproportionately affected by HIV in the United States. As Latinx sexual minority men, particularly those who are foreign-born, experience inequitable access to health services, tailored strategies to engage them for HIV prevention are urgently needed.

**Objective:**

Our study seeks to address the need for enhanced access to HIV prevention among Latinx immigrant sexual minority men. We developed and piloted a culturally sensitive technology-based campaign focused on HIV testing and pre-exposure prophylaxis (PrEP) uptake.

**Methods:**

We used a two-phase approach to assess the feasibility of community-informed social media content in engaging Latinx immigrant sexual minority men for HIV testing and PrEP use. First, we conducted three iterative focus groups with 15 Latinx immigrant sexual minority men to refine the HIV prevention content to be piloted on social media platforms. The finalized content was placed on Instagram and Facebook for 9 days in July and September 2021 to individuals who were in Washington State. Individuals who clicked on the content were directed to a website with additional HIV prevention information. Second, we conducted online surveys (n=60) with website visitors that assessed sociodemographic characteristics, barriers to HIV prevention, and HIV-related transmission risk and prevention behaviors. We conducted descriptive analyses to examine the overall profile of survey respondents and determine the feasibility of culturally informed social media content in reaching Latinx immigrant sexual minority men.

**Results:**

Overall, 739 unique users visited the website during the 9-day period when the social media content was posted on Instagram and Facebook. Our sample included 60 Latinx immigrant sexual minority men who completed the online survey. Participants’ mean age was 30.8 years and more than half (n=34, 57%) completed the survey in Spanish. A quarter of participants indicated that they were unauthorized immigrants and 57% (n=34) reported not having medical insurance. Participants reported, on average, having 6 different sexual partners in the last 6 months. Nearly a third of respondents had not tested for HIV in the last 6 months. Only about half (n=32, 53%) of respondents had used PrEP in the last 12 months.

**Conclusions:**

Community-driven social media and web-based strategies are feasible ways to engage Latinx immigrant sexual minority men who may traditionally lack access to HIV prevention information and services due to structural and social barriers. The results highlight that culturally relevant social media and web-based outreach strategies that are informed and developed by the community can reach Latinx immigrant sexual minority men for HIV prevention. Findings underscore the need to examine the effectiveness of social media content in promoting HIV testing and PrEP uptake in marginalized Latinx populations.

## Introduction

Despite significant progress in reducing the number of new HIV infections, there were more than 36,000 new HIV diagnoses in the United States and dependent areas in 2019 [1]. The vast majority of these new diagnoses occurred in gay, bisexual, and other sexual minority men, with Latinx sexual minority men accounting for nearly a quarter of all new HIV infections [1]. Latinx sexual minority men encounter significant social and structural challenges to seeking and receiving appropriate HIV prevention services [2,3]. Specifically, factors such as language barriers, immigration status, education level, and discrimination may be linked to lower uptake of HIV prevention strategies such as HIV testing and pre-exposure prophylaxis (PrEP) use in Latinx immigrant sexual minority men [2,3]. Further, with the unprecedented disruptions related to the COVID-19 pandemic, Latinx immigrant sexual minority men may be less likely to seek in-person care and information for nonurgent conditions, potentially delaying HIV testing and PrEP use. Hence, while effective strategies for HIV prevention and treatment are available, increased efforts to enhance their access and use among Latinx immigrant sexual minority men are urgently needed.

The use of eHealth and related technology has the potential to improve the reach and implementation of HIV prevention strategies for marginalized populations such as Latinx immigrant sexual minority men [4,5]. HIV prevention information delivered via online and social media platforms may offer greater privacy and confidentiality, which may increase the acceptability of accessing HIV prevention resources [5]. eHealth tools have also served as a useful mechanism to deliver information and health services when in-person activities are limited or unavailable due to public health concerns such as COVID-19 [6]. Further, the wide use and availability of the internet and social media applications have made eHealth HIV prevention approaches increasingly accessible for hard-to-reach populations [7,8]. For Latinx immigrant sexual minority men who may be reluctant about seeking health services due to stigma or fear, eHealth may address barriers and support engagement with HIV prevention resources. Yet, despite the growing number of eHealth HIV prevention interventions, there has been limited attention to the acceptability of using eHealth strategies with Latinx immigrant sexual minority men [9,10].

To address the need to tailor HIV prevention programs for specific groups, we sought to enhance understanding of the specific preferences and acceptability of using eHealth strategies for HIV prevention among Latinx immigrant sexual minority men. We conducted an initial qualitative study to examine how to appropriately use social media platforms to recruit and engage Latinx immigrant sexual minority men for HIV testing and PrEP uptake [11]. We also assessed how specific content delivered on social media platforms can address barriers to HIV testing and PrEP in this population. The findings of this study indicated that Latinx immigrant sexual minority men were enthusiastic about receiving HIV prevention information via social media platforms. Participants offered specific suggestions and preferences regarding how social media content should be developed and emphasized that messaging be inclusive, motivational, and positive [11].

Based on the results of this initial study, which are published elsewhere [11], we developed content to be piloted on social media platforms to engage Latinx immigrant sexual minority men for HIV testing and PrEP uptake. This study examines the feasibility of using the culturally tailored content on social media platforms to reach Latinx immigrant sexual minority men for HIV prevention.

## Methods

### Overview

This feasibility study used a community-based participatory approach and was conducted in Seattle, Washington in partnership with a local community-based organization that serves the lesbian, gay, bisexual, transgender, and queer (LGBTQ+) Latinx community. The first phase of the study involved three focus groups that were developed to finalize the social media content for the study. During the study’s second phase, the finalized content was piloted on social media platforms, and a web-based survey was conducted to assess whether the social media content was successful in reaching Latinx immigrant sexual minority men for HIV testing and PrEP uptake.

### Focus Groups

We developed five initial designs of the culturally tailored HIV prevention content to be piloted on social media platforms based on the preferences identified from Latinx immigrant sexual minority men. We conducted three iterative focus groups with 15 Latinx immigrant sexual minority men to further revise the initial designs according to participants’ preferences. During each focus group, participants offered suggestions to edit the content and ensure the cultural sensitivity of the designs. Participants also discussed the rationale behind their suggested changes. We finalized the designs to be piloted on social media platforms after the third and final focus group.

### Pilot Testing of the Social Media Content for Latinx Immigrant Sexual Minority Men

The social media content was piloted on social media sites in July 2021. Specifically, the content was placed on Facebook and Instagram over a 9-day period. The content was available to individuals who were on Instagram or Facebook in Washington State. Individuals who clicked on the content were directed to a website that offered additional information on HIV testing and PrEP. Specifically, the website was developed with support from the study’s community partner and offered localized and culturally relevant HIV prevention information in Spanish. Information about how and why to get tested for HIV and use PrEP were included. Additionally, contact information to make an appointment to test for HIV or learn more about PrEP were also available on the website. We conducted an online survey with 60 website visitors to assess whether the social media content was a feasible strategy for reaching Latinx immigrant sexual minority men for HIV prevention. To be eligible to complete the survey, individuals had to meet the following inclusion criteria: be 18 years or older, identify as Hispanic or Latino/a/x, male sex at birth, born outside of the continental United States, and report sex with men. All individuals consented to participate prior to completing the survey. Surveys were completed in Spanish or English based on the participant’s preference.

### Measures

Surveys were available in Spanish or English and assessed sociodemographic characteristics, barriers to HIV prevention, and HIV risk and prevention behaviors. Barriers to HIV prevention included not having health insurance and sexual orientation disclosure (whether or not individuals disclosed their sexual orientation to friends who are heterosexual, family members, employers or teachers, and health care providers). We also assessed participants’ perceptions of their community’s tolerance toward individuals who identify as LGBTQ+ (1 strongly disagree, 2 disagree, 3 neither agree nor disagree, 4 agree, 5 strongly agree). Surveys also measured the extent to which participants experienced distress related to COVID-19 (1 no distress, 10 extreme distress). HIV transmission risk behaviors were assessed by the number of sexual partners in the last 6 months, drug or alcohol use before last sex, and condomless anal sex at last sex. HIV prevention behaviors included ever testing for HIV, testing for HIV in the last 6 months, PrEP awareness, and PrEP use in the last 12 months. Descriptive analyses were used to examine the overall profile of website visitors and to determine the feasibility of using community-informed social media content to reach Latinx immigrant sexual minority men in need of HIV testing and PrEP who encounter barriers to HIV prevention.

### Ethics Consideration

The authors obtained informed consent from all study participants prior to their participation in the focus groups or web-based surveys. All study procedures were reviewed by the University of Washington Human Subjects Division and qualified for exempt status from federal human subjects regulations.

## Results

### Focus Groups

**Figure 1:**
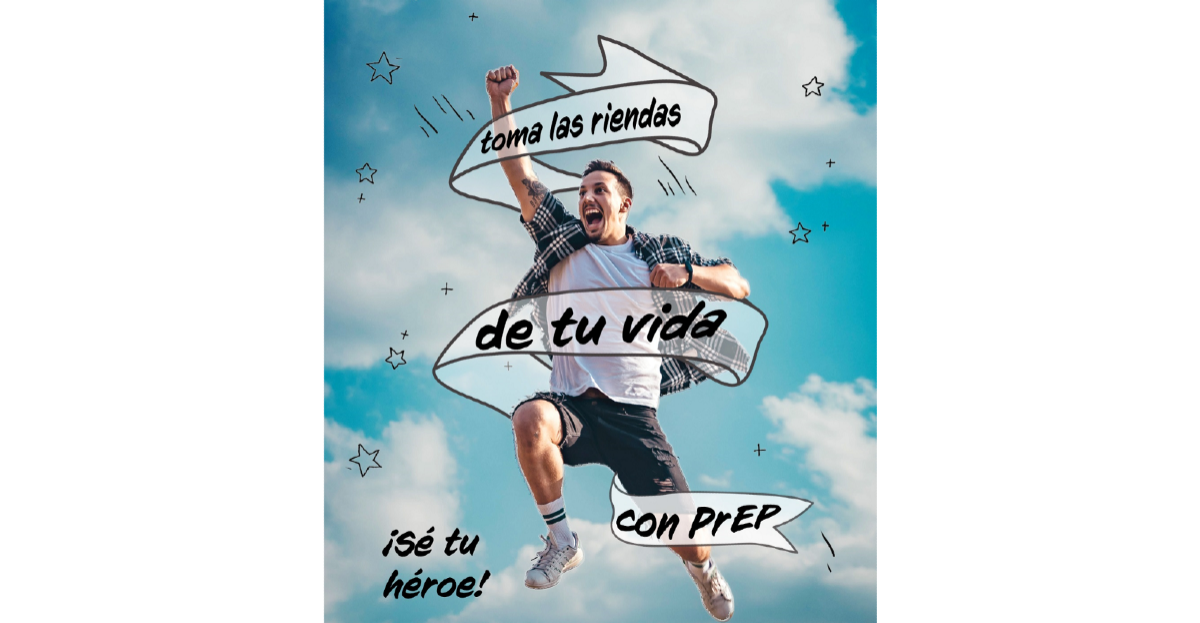
Culturally tailored social media content for HIV prevention in Latinx immigrant sexual minority men: Be your hero.

**Figure 2:**
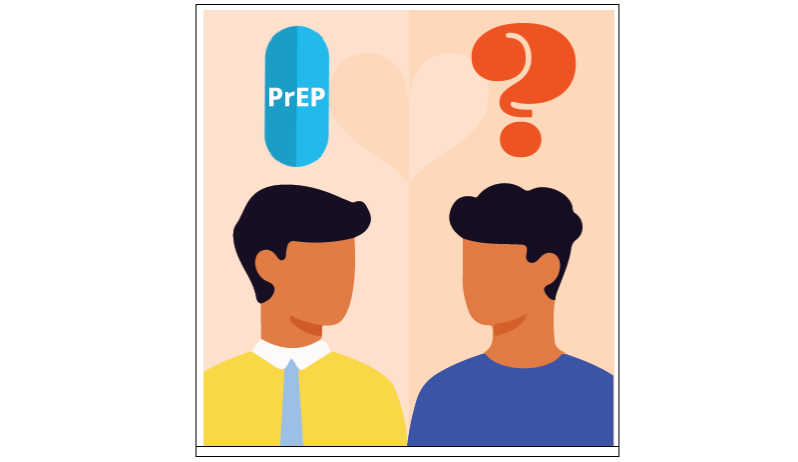
Culturally tailored social media content for HIV prevention in Latinx immigrant sexual minority men: Illustrated men. PrEP: pre-exposure prophylaxis.

**Figure 3:**
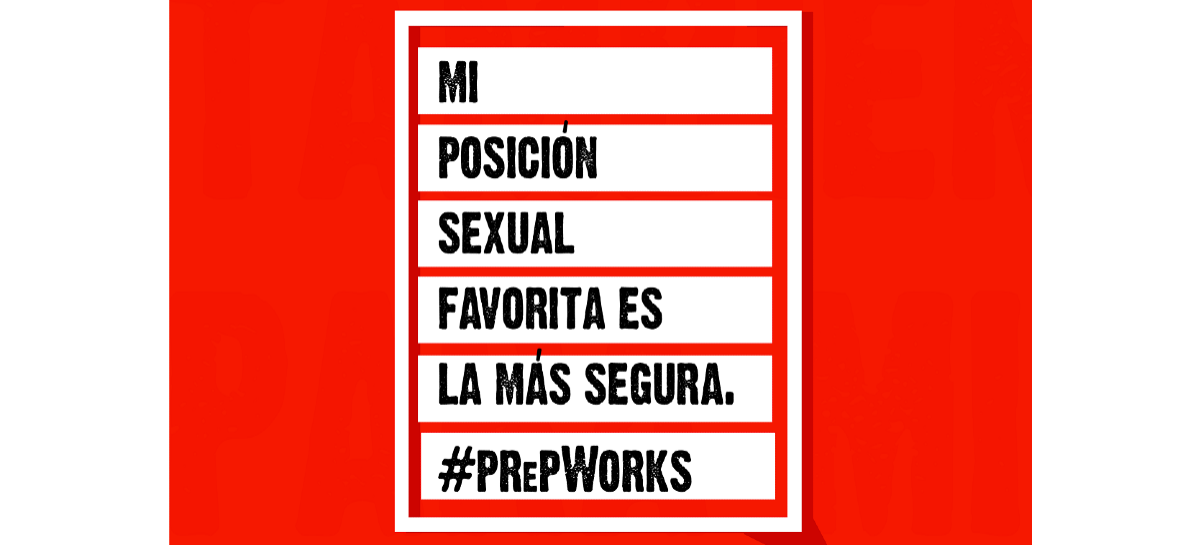
Culturally tailored social media content for HIV prevention in Latinx immigrant sexual minority men: Favorite sexual position. PrEP: pre-exposure prophylaxis.

**Figure 4:**
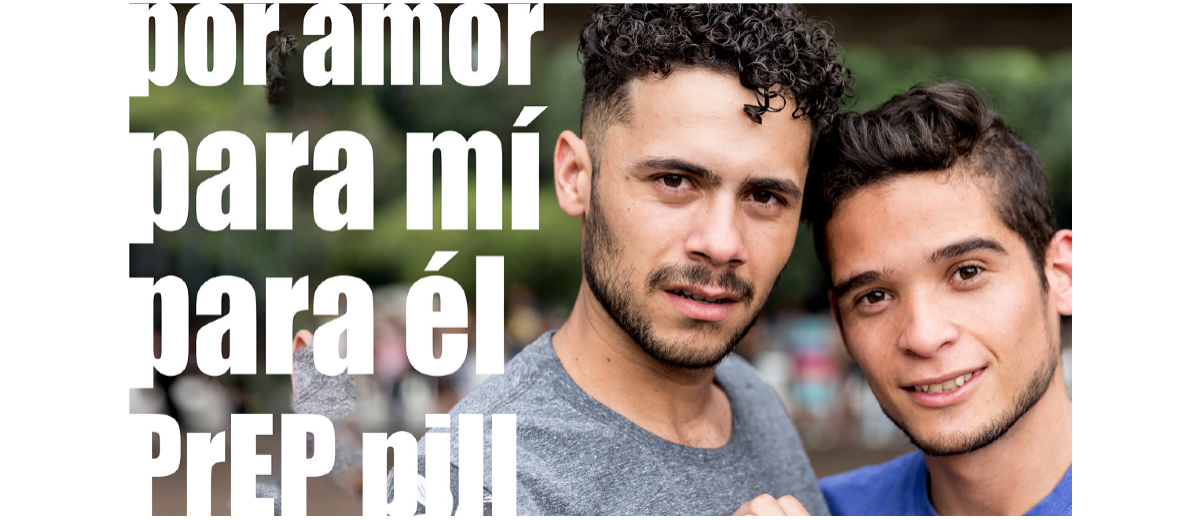
Culturally tailored social media content for HIV prevention in Latinx immigrant sexual minority men: For love. PrEP: pre-exposure prophylaxis.

**Figure 5:**
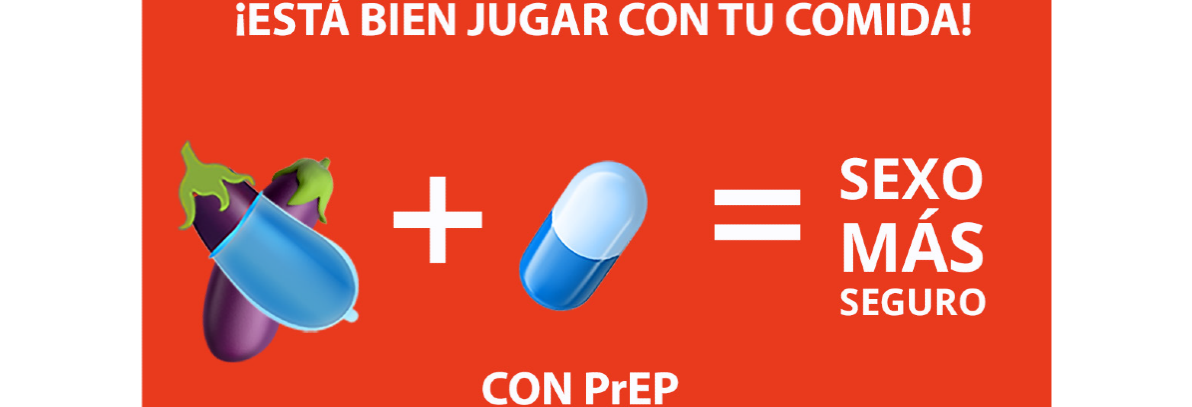
Culturally tailored social media content for HIV prevention in Latinx immigrant sexual minority men: Emojis. PrEP: pre-exposure prophylaxis.

The focus groups provided feedback on the language, imagery, and overall designs of the community-informed social media content. The initial versions of the content were revised in the following ways based on focus group feedback: photographs were changed to depict more realistic images of Latinx sexual minority men, additional colors and illustrations were integrated to highlight the empowering aspects of HIV prevention, and designs that were text-focused were included to provide more discreet ways to engage with the content. The finalized content is presented in Figures 1-5.

Figure 1 states “Take charge of your life with PrEP” and “Be your hero” in Spanish, and presents a multimedia image of a man in the sky. Figure 2 presents an illustrated image of two men and a symbol of PrEP. Figure 3 is text-focused and states, “My favorite sexual position is the one that’s safest” in Spanish with the hashtag “#PrEPWorks.” Figure 4 includes an image of two men with the words “For love,” “For me,” “For him,” in Spanish and “PrEP pill.” Figure 5 states, “It’s okay to play with your food” in Spanish and uses emojis and illustrations to demonstrate that sex with condoms and with PrEP is the “safest sex.” These five designs reflect community priorities, preferences, and needs to receiving information and services related to HIV prevention. For example, the use of “Spanglish” or both English and Spanish in the designs was encouraged as it highlights that many Latinx immigrant sexual minority men speak both Spanish and English. Additionally, participants noted that the use of humor and cheeky language and imagery were an important way to catch their attention. Overall, the content relays the positive and motivational messages sought by focus group participants.

### Characteristics of Feasibility Study Survey Respondents

There were 739 unique users who visited the website after clicking the pilot social media content on Facebook or Instagram over the 9-day period. Among the 60 Latinx immigrant sexual minority men who completed the online survey, slightly more than half (n=34, 57%) completed the survey in Spanish. Respondents’ sociodemographic characteristics are presented in Table 1.

The mean age was 31 years, and the average length of time residing in the United States was 14 years. About half of the respondents (n=32, 53%) were born in Mexico and 20% (n=12) reported having less than a high school level education. More than half of the participants (n=35, 58%) reported their religion as Catholic or Christian. A quarter of participants were unauthorized immigrants, while 17% (n=10) were legal permanent residents (Table 1).

**Table 1:** Characteristics of 60 Latinx immigrant sexual minority men who were reached by the culturally informed pilot social media content.

			Participants, n (%)
Age (years), mean (SD)			30.8 (8.2)
**Country of origin, n (%)**			
	Mexico	32 (53)	
	El Salvador	6 (10)	
	Honduras	4 (7)	
	Cuba	4 (7)	
	Puerto Rico	4 (7)	
	Other^a^	10 (17)	
Length of time in United States (years), mean (SD)			13.9 (8.5)
**Education, n (%)**			
	Less than high school	12 (20)	
	High school or GED^b^	14 (23)	
	Some college	13 (22)	
	Bachelor’s degree or higher	21 (35)	
**Employment status, n (%)**			
	Unemployed	9 (15)	
	Employed full time	32 (53)	
	Employed part time	14 (23)	
	In school	4 (7)	
	Retired	1 (2)	
**Religion, n (%)**			
	Catholic	18 (30)	
	Protestant/Christian	17 (28)	
	No religion	11 (18)	
	Other^c^	8 (13)	
	Atheist	4 (7)	
**Immigration status, n (%)**			
	Legal permanent resident	10 (17)	
	Naturalized citizen	8 (13)	
	Unauthorized immigrant	15 (25)	
	Eligible immigrant	24 (40)	
	Temporary resident or other	3 (5)	

^a^Other included Argentina, Brazil, Panama, Guatemala, Colombia, Ecuador, and Peru.

^b^GED: General Educational Development.

^c^Other included: Nondenominational or independent, spiritual but not religious, and other.

### Barriers to HIV Prevention

Select barriers to HIV prevention are presented in Table 2.

Approximately 42% (n=25) of respondents indicated that they did not have health insurance. While the majority (n=53, 88%) of participants reported disclosing their sexual orientation to their health care providers, less than three-fourths had disclosed their sexual orientation to friends who are heterosexual or to family members. About half (n=31, 52%) of respondents had disclosed their sexual orientation to employers or teachers. Participants reported an average rating of 3.5 (range 1-5) in community tolerance toward LGBTQ+ individuals. On a scale of 1 to 10 with “1” being no distress and “10” being extreme distress, respondents reported experiencing an average level of 5.0 in COVID-19–related distress.

**Table 2:** Barriers to HIV prevention and HIV transmission risk and prevention behaviors among Latinx immigrant sexual minority men (N=60).

		Participants, n (%)
**Health insurance, n (%)**		
	Yes	34 (57)
	No	25 (42)
	Unsure	1 (2)
COVID-19–related distress, mean (SD)		5.0 (2.9)
Community LGBTQ+^a^ tolerance, mean (SD)		3.5 (1.3)
**Sexual orientation disclosure^b^, n (%)**		
	Disclosed to friends who are heterosexual	43 (72)
	Disclosed to family members	42 (70)
	Employers or teachers	31 (52)
	Disclosed to health care providers	53 (88)
Number of sexual partners in last 6 months, mean (SD)		6.2 (7.1)
Drug or alcohol use behavior during last sex^c^, n (%)		18 (30)
Condomless anal sex at last sex^c^, n (%)		24 (40)
Ever tested for HIV^c^, n (%)		48 (80)
Tested for HIV in last 6 months^c^, n (%)		42 (70)
Currently using PrEP^c,d^, n (%)		28 (47)

^a^LGBTQ+: lesbian, gay, bisexual, transgender, and queer.

^b^Data presented indicates respondents who disclosed.

^c^Data presented indicates respondents who reported “Yes” to the question.

^d^PrEP: pre-exposure prophylaxis.

### HIV Transmission Risk and Prevention Behaviors

Table 2 also presents respondents’ HIV transmission risk and prevention behaviors. Respondents reported an average of 6.2 sexual partners in the last 6 months and 30% (n=18) indicated using alcohol or drugs during last sex. While the majority (n=48, 80%) of participants had ever tested for HIV, 70% (n=42) had tested for HIV in the last 6 months. Less than half (n=28, 47%) of all respondents were currently using PrEP (Table 2).

## Discussion

### Primary Findings

Our results demonstrate that the pilot social media content reached a diverse sample of Latinx immigrant sexual minority men. Specifically, we engaged Latinx immigrant sexual minority men who were undocumented, had low levels of education, and were unemployed. While these factors often present challenges to accessing HIV prevention services [3,12,13], culturally tailored social media outreach strategies may provide unique opportunities to obtain HIV prevention information and resources that are traditionally out of reach for these communities. Further, more than half of respondents identified as Catholic or Protestant/Christian, which suggests that our social media content was a feasible approach to engaging Latinx sexual minority men who are members of religious organizations. Prior research has documented that Latinx sexual minority men who are religiously affiliated may experience greater internalized homophobia, which can have detrimental effects on HIV prevention and overall health [14]. While additional studies are needed to better understand the role of religion on HIV prevention in Latinx sexual minority men [15], culturally tailored HIV prevention content delivered via social media applications may provide a potential way to engage religious Latinx communities who may be deterred to access such information due to perceptions of internalized or societal stigma.

Our pilot content was also successful in reaching Latinx immigrant sexual minority men who reported several barriers to HIV prevention, including being uninsured. Not having health insurance can lead to missed opportunities for HIV testing and limit access to preventive health services [16]. As Latinxs have the highest uninsured rate of any racial or ethnic group in the United States [17], eHealth may be a valuable tool for reaching individuals who experience systemic health inequities. eHealth may also be a feasible HIV prevention strategy among Latinx immigrant sexual minority men in diverse contexts. Despite overwhelming challenges due to the COVID-19 pandemic, Latinx immigrant sexual minority men who reported experiencing distress related to COVID-19 engaged with the tailored HIV prevention social media content. Additionally, many respondents reported feeling that their communities were somewhat intolerant of individuals who identify as LGBTQ+. As sexual minority men who face intolerance and discrimination may be less likely to use HIV prevention services, prevention services and information available through social media platforms may facilitate use among Latinx immigrant sexual minority men by creating a more acceptable environment in which to engage.

Further, a sizeable percent of respondents noted that they had not disclosed their sexual orientation to others, suggesting that many Latinx immigrant sexual minority men who were reached by the social media content may have concerns about prejudice and discrimination in their various social networks. Social media platforms and other eHealth tools can offer methods that may be more acceptable for individuals who seek to conceal their sexual orientation from specific groups and desire a discreet way to obtain HIV prevention services. Taken together, the tailored social media content was a feasible way to reach Latinx immigrant sexual minority men for HIV prevention given that its format and delivery method may overcome prior barriers to using services.

Notably, a considerable percentage of the Latinx immigrant sexual minority men reported engaging in sexual risk behaviors, including having multiple sexual partners, having condomless anal sex, and using drugs or alcohol before sex. Our results also demonstrated gaps in HIV prevention with nearly a third of respondents not having tested for HIV in the last 6 months and more than half not currently using PrEP. Hence, culturally tailored social media content is a feasible way to reach Latinx immigrant sexual minority men who not only experience barriers to HIV prevention but who also exhibit behaviors that may place them at elevated risk for HIV transmission.

### Limitations

There are several limitations to this study. First, given that this study was designed to pilot the culturally informed content on Instagram and Facebook, a comparison to Latinx immigrant sexual minority men who did not engage with the social media content was not possible. Despite not having a comparison group, the authors believe that the characteristics of survey respondents highlight the opportunity that eHealth presents for reaching Latinx immigrant sexual minority men in need of HIV prevention services. Second, while the social media content was delivered virtually, the platforms focused on reaching individuals in Washington State and its Latinx population. Therefore, the social media content may not be appropriate or feasible for reaching all Latinx immigrant sexual minority men in the United States, including other locales with different compositions of Latinx immigrant populations. Further, our survey did not capture all individuals who engaged with or were impacted by the social media content. Hence, individuals who did not complete the survey may vary from those reported in this study.

### Conclusions

Our culturally relevant social media and web-based outreach strategies that were informed and developed by the community are a feasible way to reach Latinx immigrant sexual minority men for HIV prevention. Community-driven social media and web-based content present opportunities for engaging individuals who may traditionally lack access to HIV prevention information and services. Future research may examine the effectiveness of culturally informed social media content in increasing HIV testing and PrEP use among Latinx immigrant sexual minority men.

## Abbreviations

LGBTQ+: lesbian, gay, bisexual, transgender, and queer
PrEP: pre-exposure prophylaxis
